# Computational Psychometrics Using Psychophysiological Measures for the Assessment of Acute Mental Stress

**DOI:** 10.3390/s19040781

**Published:** 2019-02-14

**Authors:** Pietro Cipresso, Desirée Colombo, Giuseppe Riva

**Affiliations:** 1 Applied Technology for Neuro-Psychology Lab at IRCCS Istituto Auxologico Italiano, Via L. Ariosto 13, 20145 Milano (MI), Italy; giuseppe.riva@unicatt.it; 2 Department of Psychology of the Catholic University, Largo Gemelli 1, 20100 Milano (MI) and Applied Technology for Neuro-Psychology Lab at IRCCS Istituto Auxologico Italiano, Via L. Ariosto 13, 20145 Milano (MI), Italy; 3 Department of Basic Psychology, Clinic and Psychobiology, Universitat Jaume I, Av. Sos Baynat, s/n, 12071 Castellón, Spain; dcolombo@uji.es

**Keywords:** computational psychometrics, psychophysiology, psychological stress, acute mental stress, acute time-limited stressors, Stroop color word task, arithmetic task

## Abstract

The goal of this study was to provide reliable quantitative analyses of psycho-physiological measures during acute mental stress. Acute, time-limited stressors are used extensively as experimental stimuli in psychophysiological research. In particular, the Stroop Color Word Task and the Arithmetical Task have been widely used in several settings as effective mental stressors. We collected psychophysiological data on blood volume pulse, thoracic respiration, and skin conductance from 60 participants at rest and during stressful situations. Subsequently, we used statistical univariate tests and multivariate computational approaches to conduct comprehensive studies on the discriminative properties of each condition in relation to psychophysiological correlates. The results showed evidence of a greater discrimination capability of the Arithmetical Task compared to the Stroop test. The best predictors were the short time Heart Rate Variability (HRV) indices, in particular, the Respiratory Sinus Arrhythmia index, which in turn could be predicted by other HRV and respiratory indices in a hierarchical, multi-level regression analysis. Thus, computational psychometrics analyses proved to be an effective tool for studying such complex variables. They could represent the first step in developing complex platforms for the automatic detection of mental stress, which could improve the treatment.

## 1. Introduction

Mental stress is an important factor potentially affecting mental and physical functions. According to classical theories, stressors can be defined as challenging events requiring physiological and behavioral responses that are aimed at reinstating homeostasis. Effective coping strategies involve a rapid response that also has to be efficiently terminated afterward. If the response to the stressor is inadequate, the biological costs may become too high [1]. 

In the last half century, several studies have demonstrated the role of mental stress in many disorders, like cardiovascular diseases, abnormal cognitive functioning, or psychiatric disorders [2,3]: For instance, the inability to cope with stressors has been associated with the hypersecretion of corticosteroids and with an increased risk of depressive onset [1]. In addition, early and prolonged exposure to stressors has been shown to affect neurodevelopment, involving both neurobiological and neuroendocrine aspects, and cognitive, social. and emotional domains [4]. Early life stress in turn translates to increased psychological vulnerability and augmented risk for psychiatric disorders [5]. Concerning everyday life, acute mental stress has been proven to reduce performance, especially in the workplace [6]. According to these premises and considering the important repercussions on mental health, it is important to further study and understand mental stress and its effect on everyday life. 

Acute mental stress influences the Autonomous Nervous System. The use of psycho-physiological instruments in the assessment of acute mental stress is becoming therefore quite common due to the unobtrusiveness and non-invasiveness of sensors used to detect physiological signals [7] and the development of validated measures, like heart rate variability (HRV) [8,9]. Psychophysiological measures have been indeed validated as biological markers of acute and chronic stress [9,10]. Interestingly, these measures not only can identify short-term variations to temporary stimuli, but can also provide strong indications of dysfunctions in the long run. 

An acute, time-limited stressor can have short-term effects on cognitive and emotional processes associated with planning an action [10,11]. Specifically, an acute stressor produces an immediate response in the sympathetic nervous system due to the release of noradrenaline from widely distributed synapses, causing increased blood pressure and release of adrenaline from the adrenal medulla, which in turn induces peripheral vasoconstriction, accelerated heart rate, and muscular contractions, among other adverse effects [12,13].

Unfortunately, stressors tend to be repeated over time in normal individuals’ lives. This can be due to several factors, e.g., the challenges and pressures associated with the current society and one’s daily work schedule, unexpected problems, traumatic situations, and personal perceptions of oneself and others [14,15]. When acute stressors become a customary component of life, the immune system, the cardiovascular apparatus, and the nervous system may suffer potentially detrimental consequences [16,17,18]. 

Since acute mental stress increases the risk of developing chronic stress, acute mental stressors must be studied and understood thoroughly, especially with reference to their effects on body and mind.

As reported in a recent review by Castaldo and colleagues [19], short-term HRV analysis has been used in several studies to assess acute mental stress. In their illuminating review and meta-analysis, they reported the common trends in using HRV indices to evaluate stressors. More specifically, HRV at high frequencies resulted in significant depression during acute mental stress, further demonstrating a significant autonomic balance shift during acute mental stress towards the sympathetic activation and the parasympathetic withdrawal. Interestingly, half of their selected studies adopted two specific acute mental stressors: The Stroop Color World Task and the Arithmetical Task [20,21,22,23,24,25]. During the Stroop Color Word Task, the participants are required to name the colors of the words presented on a screen (green, yellow, orange, red, blue, purple), which can be congruent or incongruent with the meaning of the written word. This task is considered a reliable and valid method to evaluate acute mental stress and to induce a moderate level of transitory physiological modifications [26,27]. On the other hand, the Arithmetical Task requires performing arithmetical operations by subtracting a prefixed quantity (typically 17) from a starting number (typically 1013). The Arithmetic Test is considered a standard moderate acute stressor, and it is often used in physiology to detect changes in the autonomic nervous system [28].

Unfortunately, the existing studies have considered each index separately; thus, to date, only univariate statistical analyses have been conducted to assess the stressors. On the contrary, multivariate analyses, especially computational analyses, relies on the discriminative knowledge of all the variables considered as a whole or as specific subsets. Psychophysiological correlates reflect elicited emotions, and they represent variations in the central and peripheral nervous system. Thus, they must be considered in more complex models rather than only univariate analysis to identify the effect of a stressor more accurately [9,29,30,31,32]. With this consideration in mind, we provided evidence of the effectiveness of the two acute stressors (Stroop Task and Arithmetic Task) by means of computational analyses. 

On the other hand, the price of CE-marked medical devices has dropped significantly, along with a wide inclusion of biosensors in wristbands or other wearable devices [33,34,35]. An example is the wrist photoplethysmography that has been used to record blood volume pulse (i.e., BVP, an analog of electrocardiogram). Practically, the use of wearable sensors for both clinical and leisure uses has been increasing. Such sensors are becoming more technologically advanced and provide excellent sampling rates, acceptable precision, and effective artifact removal by algorithms that are embedded in the firmware [33,36,37,38,39]. Nevertheless, some problems with recording the HRV using commercial biosensors are related to the sampling rate, which according to the standard HRV guidelines, needs to be at least 100 Hz [9]. of the sampling rate of commercial HRV sensors, such as for the Apple Watch, is about 100 Hz. Moreover, the manufacturers have considered the consumption of battery when computing the indices in the biosensor firmware, allowing only the indices to be transferred to the potential application instead of the raw data. The use of computational algorithms for acute, time-limited stressors that we assessed can provide a practical understanding and ready solution for the effective detection of these important variables in our daily lives.

## 2. Methods

### 2.1. Participants

The participants in the study consisted of 60 healthy students (30 males and 30 females) with the mean age of 21.2 (SD 2.25) ranging from 19 to 25. They were requested not to drink caffeine or alcohol and not to smoke prior to the experimental test in order to avoid any effects of these substances on the central autonomic nervous system. All the experiments were conducted in accordance with the Declaration of Helsinki and approved by the Institutional Review Board of Istituto Auxologico Italiano. Written informed consent was obtained from each participant.

### 2.2. Procedures

The participants who met the experimental criteria were contacted via email and/or telephone to schedule a meeting. The researcher assisted them during the sessions, maintained a neutral tone of voice, and maintained neutral behavior while the participants were being exposed to the experimental stimuli. The participants were asked to sit in front of a computer, and they were told about the general goals of the research, the procedures to be used, and the concerns associated with study involvement. The broad functions of the electrodes were explained relative to their use in the collection of the psychophysiological indices. The researcher attached the sensor electrodes in the following order. The first sensor was the thoracic respiration belt. Two skin conductance adhesive patches were then applied to the left palm. Last, the blood volume pulse (BVP) sensor was placed on the top of the index finger of the left hand. All participants were right-handed (without a history of switching the dominant hand during their lifetimes). When the subject indicated that he or she was comfortable, the researcher asked her/him to remain still during the presentation of the stimuli in order to avoid artifacts in signal acquisitions as a result of movements. At the end of the experimental session, the experimenter helped the participants remove all of the electrodes and patches and explained the aims of the experiment and the scientific rationale for using the stimuli.

### 2.3. Experimental Stimuli

A four-minute baseline session was conducted with all participants to establish a stable reference. The order of the application of the stimuli was randomized. The sessions included: (1) a four-minute relaxation period, i.e., panoramic slide show, (2) an acute time-limited stressor, i.e., the four-minute Stroop Color Word Task (SCWT), simply indicated as “Stroop” in the analyses, and (3) a four-minute arithmetic task (AT), simply indicated as “Arithmetic” in the analyses. The relaxation session comprised a series of panoramic photographs that were validated by Mauri and colleagues [40]. In the Stroop Color Word Task, the participants were required to name the colors of the words that were congruent or incongruent with the words’ meaning [20,40]. In the Arithmetic task, the participants were required to subtract 17 from 1000, subtract 17 from the result, and continue doing so until the end of the 4-minute session. They were asked to answer as accurately as possible [21].

#### 2.3.1. Recording of the Physiological Signals

The data on the autonomic nervous systems were collected by measuring three physiological responses, i.e., Blood Volume Pulse, Galvanic Skin Response, and Respiration. These responses were acquired by means of a Procomp Infinity device from Thought Technology, and Biograph Infinity 5.0.2 software was used to record them. The responses were then processed with custom software developed using MATLAB 7.10.0 (R2010a) (The Mathworks, Inc.; Natick, MA, USA). Every channel was acquired synchronously at 2048 Hz and extracted at 256 Hz for computation of indices.

#### 2.3.2. Psychophysiological Signal Processing

Cardiovascular and respiratory activities were monitored to evaluate both the voluntary and autonomic effect of respiration on heart rate. We analyzed the Inter-Beat Interval (IBI) extracted from the Blood Volume Pulse sensor, a measure equivalent to the RR peaks interval extracted from the electrocardiogram; respiration (from a chest strip sensor); and their interactions. Inter-beat interval (IBI, following also RR) was transformed into an estimate of heart rate (HR) as well as the pulse amplitude (BVP Amplitude), both of which represent the relative increase in blood volume. The two indexes used to represent the heart rate measurements from BVP were the means of HR (in beats per minute) and RR mean (60,000/HR). According to the guidelines of Task Force of the European Society of Cardiology and the North American Society of Pacing and Electrophysiology, typical temporal and spectral Heart Rate Variability (HRV) indices can be extracted to evaluate the response of the autonomic nervous system [9,40]. As the temporal domain measure of the variability of heart rate, we calculated the standard deviation (SDRR) using the BVP IBI and the standard deviation of the average beat-by-beat heart rate (SDHR). The third measure of HRV in the temporal domain was the BVP amplitude, which represents the relative increase in blood volume caused by the heart’s contracting (vasoconstriction), and it displays the moment-by-moment HRV, thereby providing significant insight into individual’s emotional responses. For the frequency domain, spectral analysis was performed using Fourier spectral methods. In particular, Standard Heart Rate Variability (HRV) spectral-method indexes and similar indexes were used to evaluate the response of the autonomic nervous system. We calculated the magnitude of the peak frequency (also indicated as RR peak frequency) in the power spectrum. The rhythms were classified as very low frequency (VLF < 0.04 Hz), low-frequency (LF, between 0.04 and 0.15 Hz), and high frequency (HF, from 0.15 to 0.5 Hz) oscillations. This procedure also allowed us to calculate the LF/HF ratio, a well-known sympathovagal balance index.

The respiration signal was filtered to produce a smooth sinusoidal signal [40,41]. Respiration Period index represents the peak-to-peak time (maximum-to-maximum distance of the sinusoid), which allowed us to compute the Respiration rate that corresponded to the breaths per minute (BPM). Additionally, the tidal volume of the air moving in and out of the lungs during breathing was measured to obtain respiratory amplitude. Respiratory amplitude index represents the peak-to-peak amplitude as the vertical Manhattan distance between the peak (highest amplitude value) and the trough (lowest amplitude value).

The interaction between cardiovascular and respiratory activity can also be considered using the Respiratory Sinus Arrhythmia (RSA) index [9,40,41]. HR Max-HR Min is the peak-to-trough difference in heart rate that occurs during a full breath cycle. This metric is affected by RSA and is generally described as a measure of vagal tone (vagus nerve activity). High values of HR Max-HR Min represent high vagal tone.

Skin Conductance (SC) and Skin Resistance (SR) are units of electrodermal activity that are expressed as either conductance (microsiemens) or resistance (microohms). SC reflects a fairly slow physiological process, and it can be sampled at 32 Hz without distortion. The signal that we considered was expressed in microsiemens. To calculate the mean index of SC, we considered the mean of the sampled signal after the removal of artifacts. 

#### 2.3.3. Statistical Analyses

First, we analyzed the data using STATA MP-Parallel Edition (StataCorp LP, College Station, TX, USA) Release 14.0, SPSS (IBM Corporation, Armonk, NY, USA), Release 21, and JASP, Release 0.7.1.4 [42]. Conditions were compared using repeated measure analysis of variance (rmANOVA). A significant Mauchly’s test of sphericity at 0.05 p level indicated that the assumption of homogeneity of covariances was violated, so we adjusted the F-test in terms of the degree of freedom by using the Greenhouse-Geisser test [43,44]. The corrected p-values were reported accordingly. The paired conditions were compared using the pairwise comparison with adjusted alpha level to avoid an inflated type I error rate when making multiple statistical comparisons using the Bonferroni correction.

#### 2.3.4. Computational Analyses

Computational analyses were done using Python 3.4 with the Orange 3.3.5 data mining suite, which was available free in the open source code (https://github.com/biolab/orange3) and from which it is possible to see all of the algorithms used in the article. In particular, a stratified, 10-fold cross-validation was done using the following methods [45,46], i.e., (1) Logistic Regression classification algorithm with ridge regularization; (2) Random Forest classification using an ensemble of decision trees; (3) Support Vector Machine (SVM) to map inputs to higher-dimensional feature spaces that best separate different classes; and (4) Naïve Bayes, a probabilistic classifier based on Bayes’ theorem. As stated before, all the algorithms used were available in the open source code and documentation related to them can be found in the Scikit user guide, which provides a detailed explanation of all the algorithms used in the study, including rank calculation, classification tree, and learners (http://scikit-learn.org/stable/user_guide.html).

## 3. Results

In the first analysis, we used classical null hypothesis significance testing (NHST) with a repeated measure design using rmANOVA and pairwise comparisons. The idea was to determine whether our three conditions (Relax, Stroop, and Arithmetic) differ and subsequently compare the pairs (Relax vs. Stroop, Relax vs. Arithmetic, and Stroop vs. Arithmetic) to identify specific differences. By using the psychophysiological indexes computed for each epoch condition, we obtained the descriptive statistics reported in Table 1. The rmANOVA univariate tests showed statistical significance (Table 2) and pairwise comparisons (Table 3) confirmed the differences between Relax and the two stressors in all measures (excluded LF/HF), but the statistical evidence in differentiating the two stressors from each other was not evident.

The NHST revealed univariate differences in psychophysiological correlates of Relax conditions with respect to the stressors. However, it was not possible to state which of the two stressors was the more powerful discriminating acute time-limited stressor from Relax.

**Figure 1:**
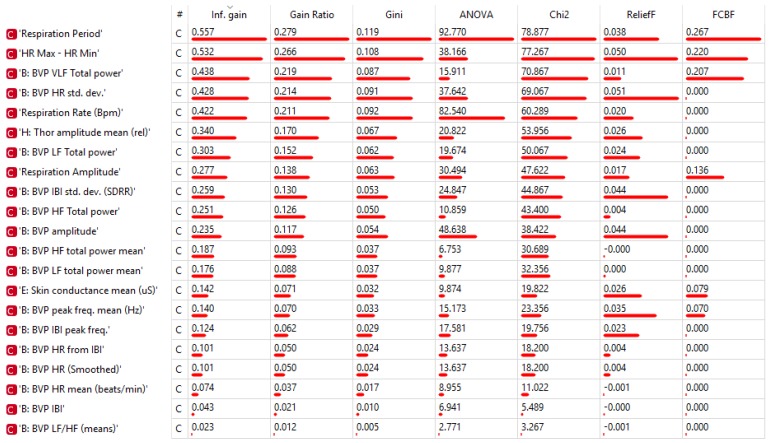
Ranks scoring algorithms results.

**Figure 2:**
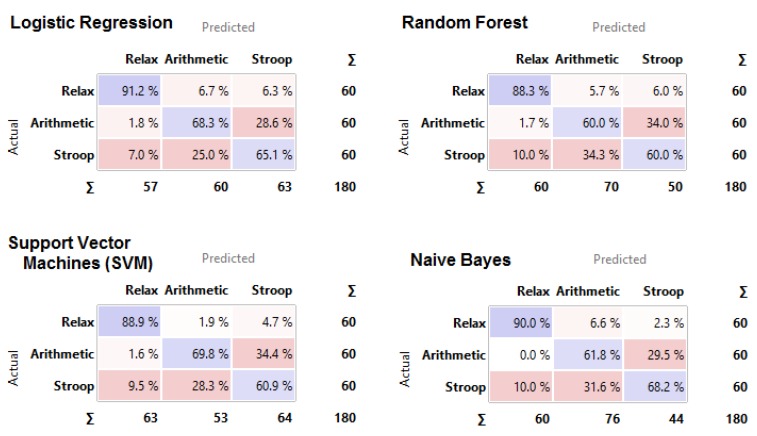
Confusion matrixes for the four classification methods.

**Figure 3:**
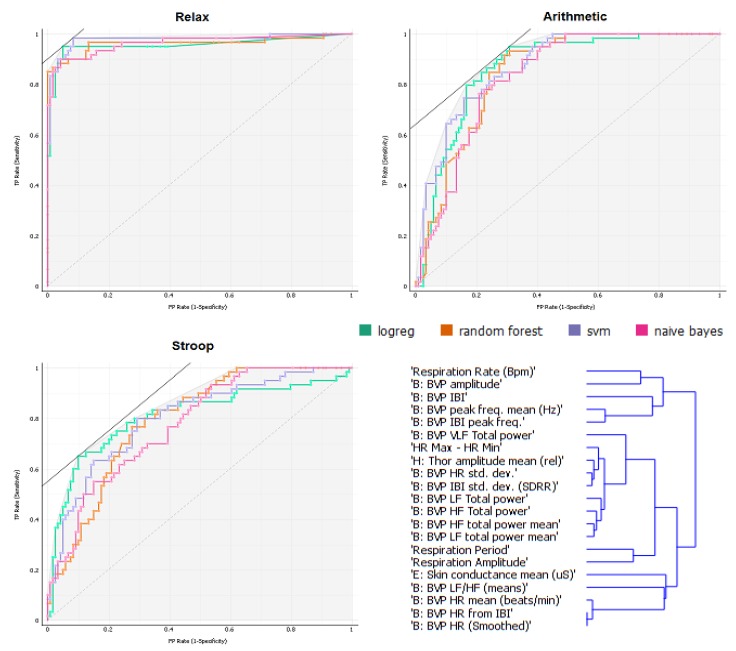
ROC Analyses for the three conditions and the hierarchical map.

**Figure 4:**
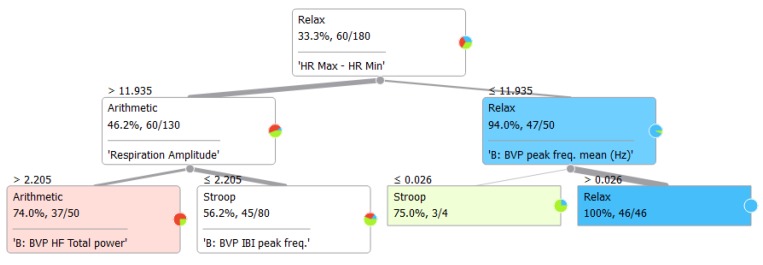
Classification Tree based on the Gini index (measure of dispersion).

To collect more information regarding this issue, we conducted computational analyses using a stratified 10-fold cross-validation with the indices ranked as shown in Figure 1 (for additional information about the rank scoring algorithms that were used for the Python computation, please see http://docs.orange.biolab.si/3/data-mining-library/reference/preprocess.html). The results showed a precision between 69.4% and 74.9% (Table 4) with most of the loss due to predicted Relax when actual was Stroop task, with an error ranging from 7% to 10%, as highlighted in the confusion matrices (Figure 2). An additional analysis of the classification is shown in Figure 3, where the true positive rate (sensitivity) is plotted against the false positive rate (specificity) and the hierarchical classification of measures. Figure 4 shows the classification tree (http://scikit-learn.org/stable/modules/tree.html) that was developed.

**Figure 5:**
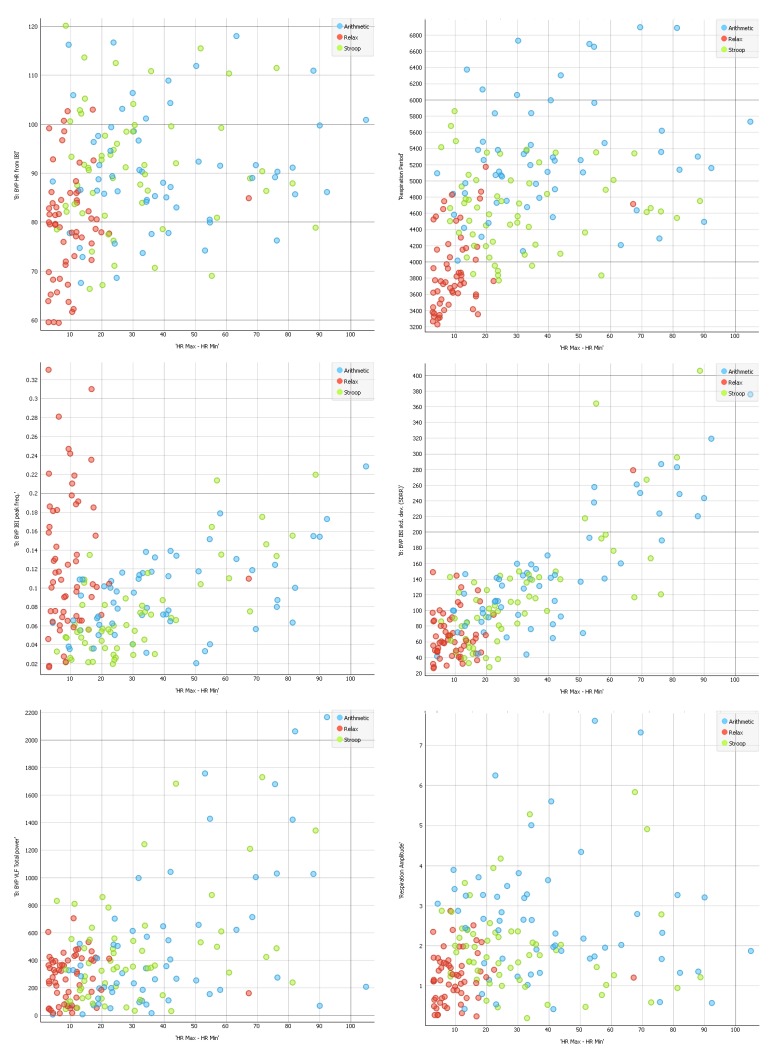
Scatter plots for HR Max–HR min over other cardiorespiratory measures.

**Figure 6:**
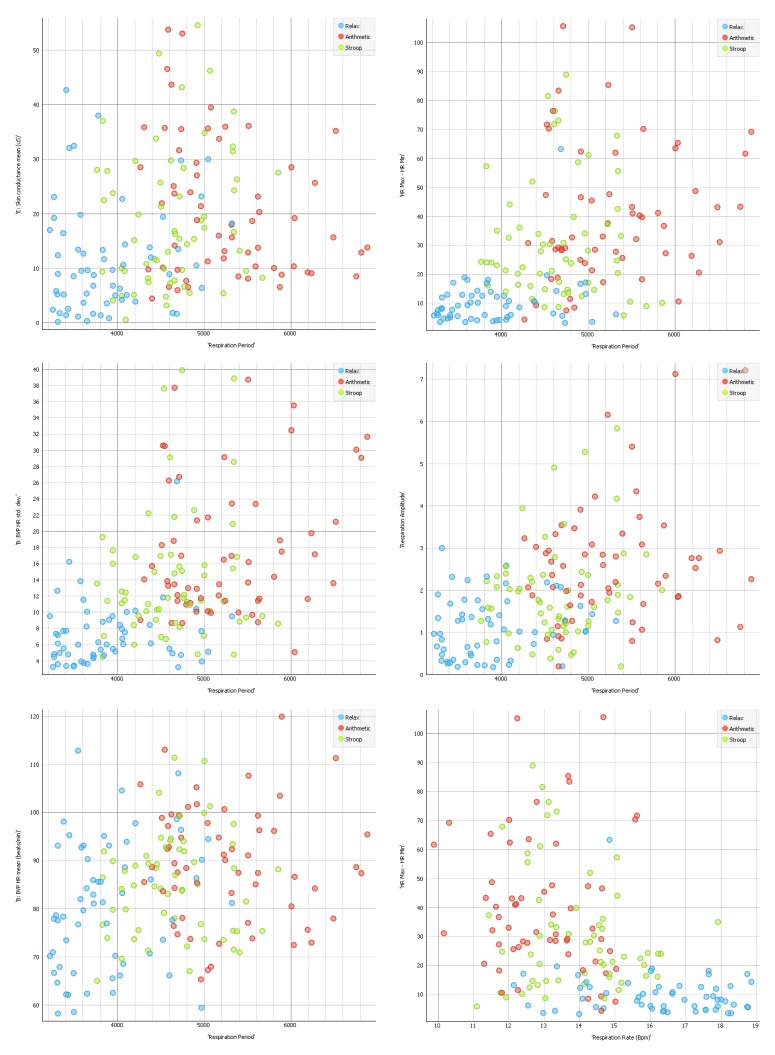
Scatter plots for Respiration over other cardiorespiratory measures.

**Figure 7:**
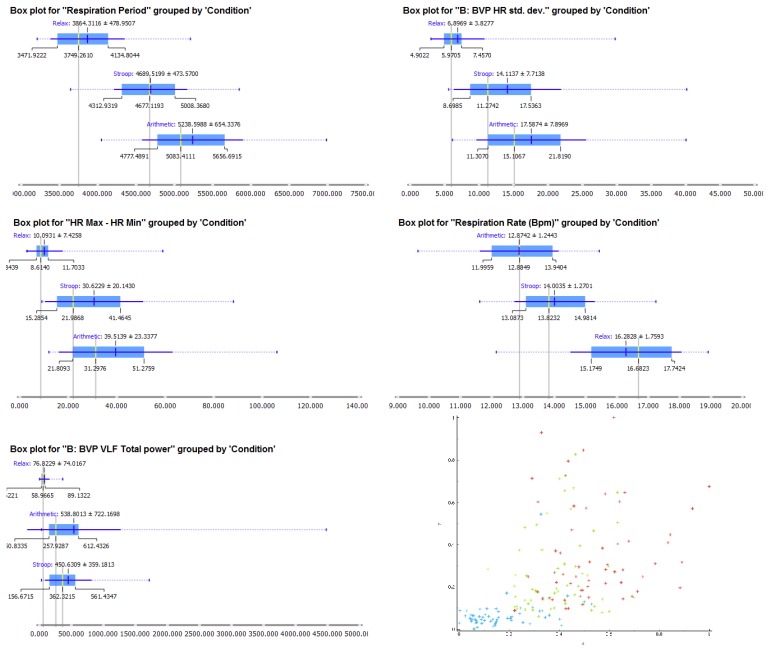
Box plot for the first five measures ranked for classification (Figure 1) and a scatter plot of all the measures standardized between zero and one and averaged among them.

A multi-level regression analysis was used to estimate the effect of HRV and respiratory indices on a possible global stress level measured through RSA. This statistical approach, also known as the Hierarchical Linear Model (HLM) [50], was chosen because the within-subject data were collected at three time points (the conditions), determining a nested data structure. The log-likelihood ratio test was then used to determine which model provided the best fit to the data. The results of the multi-level regression analysis for the RSA index (HR Max–HR min) are shown in Table 5 and plotted in Figure 5. (Respiration period is plotted in Figure 6 and Figure 7 for exploration.) The index was predicted successfully by several HRV and Respiration indices, with SDRR, Respiration Period, HR, RR peak frequency, and VLF models providing the best-fit estimates of the parameters. The model accounted for a Quasi Likelihood under Independence Model Criterion (QIC) of 11482.457 (10827.157 with 11 measures) and a Corrected Quasi Likelihood under Independence Model Criterion (QICC) of 11475.866 (10812.777 with 11 measures). The Wald χ^2^ is shown in Table 5 with the Beta slopes of the regression and the statistical significance levels.

## 4. Discussion

The goal of this study was to quantitatively analyze psychophysiological measures during mental stress. Consistent with a great number of studies [19], we adopted the Stroop Task and the Arithmetic Task to induce mental stress. We computed several indices using signal processing data analysis. Cardiovascular activity, respiratory activity, and their interactions were measured by heart rate variability indices in the time and spectral domains and respiratory sinus arrhythmia. In addition, we considered skin conductance as an index of sympathetic activity.

Castaldo and colleagues [19] reported the expected value for psychophysiological HRV indices during acute mental stress, considering each one separately with a statistical univariate approach. In this sense, our results, except for HF index, confirmed the same trends highlighted by Castaldo and colleagues in the final meta-analysis that included all the studies in the calculation [19]. 

In particular, the cardiovascular activity showed an increased physiological activation by increasing the heart rate (HR) and decreasing its inverse expressed in RR peaks distances in millisecond (RR mean). Temporal short term heart rate variability decreased by increasing the standard deviation of the heart rate (SDHR), corresponding to a decrease in the SDNN, RMSSD, and pNN50 indices reported by Castaldo in the meta-analysis [19]. 

Regarding the frequency domain features, Castaldo reported that during acute mental stress, most studies showed increased sympathetic activation, as measured by low-frequency index (LF), and a decreased parasympathetic activation, as measured by high-frequency index (HF). The consequent sympathovagal balance is just the ratio between these two indexes (LF/HF) and reflects their trends accordingly. In our study, we found also an increase in sympathetic activity with a significantly higher LF during acute mental stress compared to relax condition. On the other hand, we found a different trend in HF, indicating also an increase of parasympathetic activity. This result is not actually surprising. In fact, Castaldo reported that Vuksanovic and colleagues noted the same trend in a 2007 study with an arithmetic task that was exactly the same that we used [19]. This effect could be due to an engagement that would produce a higher level of parasympathetic activity expressed with high frequencies (HF), as has also been reported in other studies [40,41]. In the frequency domain, we also calculated very low frequencies, which showed the same trend as did high frequencies, as expected. We calculated also the peak frequency index that we suggest integrating into further studies on acute mental stress.

Castaldo and colleagues showed the trends of each HRV index during acute mental stress tasks [19]. It seems that a huge gap exists in the literature by not considering the respiration indexes and the role of the respiration in heart rate variability. Since respiration is a very easy signal to record, we included it in the analysis of our 60 participants, discovering that due to its important properties, it can be a useful tool to explore deeply in the future. Our results showed a specific pattern of respiration during acute mental stress tasks. In particular, acute mental stress affects respiration by increasing respiration amplitude and respiration period, which means fewer breath per minute but deeper respiration. Mauri and colleagues also showed increased respiration period during acute mental stress [40]. 

Recording a respiration signal we are also able to compute another important index, namely the respiratory sinus arrhythmia RSA, which shows a heart rate variability in synchrony with respiration. RSA is an indirect measure of vagal tone, reflecting the way in which vagus nerve regulates emotional functions. The RSA index that we used (HR Max–HR Min) is one possible measure of vagal tone [40,41]. Our results showed a decreased vagal tone during acute mental stress. Moreover, this index appears to be effectively segregate the data into the three conditions. Indeed, HR Max-HR Min, RR peak frequencies, and Respiration Amplitude are able to predict relax or the two stress tasks (Figure 4), but they are not hugely reported in the literature. As such, they should be considered more consistently in future studies on acute mental stress.

Our results are consistent with the general values and directions of the current scientific literature on the effects of acute, time-limited stressors on HRV indices, as reported by Castaldo and colleagues [19]. However, our study went well beyond the current studies in the two aspects described below.

First, different stressors have never been compared before. In fact, although some studies evaluated different types of stressors, they never compared them [19]. In this respect, our study highlighted the substantial differences between Stroop and Arithmetic tasks and clarified how different types of mental stress induce different psychophysiological reactions, even if in the same direction. In particular, the Stroop Task appeared to be more related to short attentional processes than to mental stress probably because involving a cognitive task also resulted in engagement states failing to be different from Relax for such aspects [40]. However, even if less engaging, the Arithmetic task produced a physiological pattern indicating acute mental stress: this task can be therefore considered more appropriate for determining the associations between acute mental stress and short-term psychophysiological reactions, which is consistent with the literature overview [19,40,41]. Stroop Task is supposed to involve executive functions, as subjects are required to act differently from their usual tendencies (not read the word, but the color). Neuroimaging studies have shown that this task activates the anterior cingulate gyrus, the dorsolateral PFC, and the parietal area [41,51,52,53].

On the other hand, we included respiration signal recording in our study. Indeed, although this is a very simple signal to record, studies inquiring respiration indices during acute mental stress are lacking and our contribution represents the first step toward a deeper understanding of the influence of respiration on mental stress in general. Respiration has the advantage to be under the direct control of conscious states, which means that we are able to modulate respiration rate and amplitude at our convenience. This study wants to promote the use of respiration techniques to treat mental stress. However, the extent to which we can invert the process needs to be investigated. In fact, from our study, it seems clear (even if to deeper investigate) that higher acute mental stress is associated with higher respiration amplitude and period; nevertheless, the extent to which reducing respiration amplitude and period can reduce the mental stress needs to be examined accurately, and this would make totally sense according to HRV biofeedback techniques wider used. In this sense, the further investigation of the RSA is an important future challenge.

In our study, we also used computational techniques based on open-source algorithms developed in Python that can classify mental states based only on psychophysiological measures. This aspect poses new challenges for the automatic recognition of stress using machine learning algorithms, which can be implemented in advanced platforms for the recognition of mental stress. In fact, these platforms would require a training dataset based on the subject’s signals in order to work properly. Our study demonstrated that the Arithmetic task rather than Stroop was a better first task for a machine learning training set. Interestingly, the Arithmetic task was also practically easier when compared to the Stroop, which required a monitor showing colored words. Moreover, the Arithmetic task could be implemented easily in a mobile App, for example, as a text message or a recorded message with simple instructions.

The classification tree (Figure 4) and the hierarchical regression analysis (Table 5) highlighted the importance of combining cardiovascular and respiratory aspects by the means of the RSA indexes and also through the Respiration period (see Figure 6). This result underlines the importance of the respiratory signal, which is often neglected in mental stress research. It is especially important when the aim is to collect data during daily activities by the means of wearable sensors and when the inclusion of a strip can be difficult. For unobtrusiveness, BVP and GSR can be detected by a wrist sensor, and a belt over the clothing can be used to detect the respiration signal. The results of our study suggest that a respiratory strip should be included in mental health studies, especially when it is important to detect short-duration events.

Our findings have implications for the computational psychometrics field [54,55]. The results of this study showed a significant potential of new computational techniques. In particular, the challenge of providing automatic feedback to users and patients can allow the development of new forms of treatment based on psychophysiological sensors. Indeed, biofeedback [56], a validated treatment for mental stress based on heart rate variability [57] or other physiological indexes [58,59,60], can be implemented in applications for the management of mental stress by using the same sensors that were used to assess stressful events. Currently, just one platform has been developed for this purpose by Gaggioli and colleagues in a block-randomized controlled trial [33], but the application of such sensors could be extended to other uses. Our findings could be extended to the implementation of an actual platform as an additional step forward in the treatment of mental stress.

## Figures and Tables

**Table 1:** Descriptive Statistics.

Measure	Condition	N	Mean	Std. Dev.	Std. Error
**Respiration Amplitude**	Relax	58	1.041	0.633	0.083
	Stroop	58	1.863	0.988	0.130
	Arithmetic	58	2.622	1.477	0.194
**Respiration Period**	Relax	58	3867.726	486.562	63.889
	Stroop	58	4695.291	479.727	62.991
	Arithmetic	58	5248.448	661.329	86.837
**Respiration Rate (BPM)**	Relax	58	16.266	1.785	0.234
	Stroop	58	13.984	1.283	0.168
	Arithmetic	58	12.852	1.254	0.165
**BVP Amplitude**	Relax	58	9.814	5.488	0.721
	Stroop	58	3.824	2.674	0.351
	Arithmetic	58	3.669	2.364	0.310
**RR mean**	Relax	58	772.724	111.797	14.680
	Stroop	58	704.346	95.495	12.539
	Arithmetic	58	705.889	97.820	12.844
**HR**	Relax	58	79.688	11.251	1.477
	Stroop	58	90.207	11.826	1.553
	Arithmetic	58	90.673	12.395	1.628
**RR peak frequency**	Relax	58	0.128	0.070	0.009
	Stroop	58	0.068	0.047	0.006
	Arithmetic	58	0.092	0.042	0.006
**HR Max–HR min**	Relax	58	10.093	7.491	0.984
	Stroop	58	30.623	20.319	2.668
	Arithmetic	58	39.514	23.542	3.091
**SDHR**	Relax	58	6.843	3.872	0.508
	Stroop	58	14.090	7.846	1.030
	Arithmetic	58	17.385	7.880	1.035
**VLF**	Relax	58	73.494	70.748	9.290
	Stroop	58	438.394	352.913	46.340
	Arithmetic	58	470.880	512.707	67.322
**LF**	Relax	58	226.405	263.204	34.560
	Stroop	58	854.887	997.451	130.972
	Arithmetic	58	1273.526	1177.104	154.561
**HF**	Relax	58	263.750	575.001	75.501
	Stroop	58	908.667	1986.858	260.887
	Arithmetic	58	1309.563	1915.539	251.523
**LF/HF**	Relax	58	1.804	1.337	0.176
	Stroop	58	1.990	1.658	0.218
	Arithmetic	58	1.426	1.036	0.136
**Skin Conductance (SC)**	Relax	58	11.603	10.600	1.392
	Stroop	58	19.643	12.781	1.678
	Arithmetic	58	20.893	12.675	1.664

**Table 2:** rmANOVA Univariate Tests (Greenhouse-Geisser corrected).

Measure	df	F	Sig.	Partial η^2^
**Respiration Amplitude**	1.418	85.732	<0.001	0.601
**Respiration Period**	1.744	102.223	<0.001	0.642
**Respiration Rate (BPM)**	1.635	86.097	<0.001	0.602
**BVP Amplitude**	1.080	106.903	<0.001	0.652
**RR mean**	1.353	33.355	<0.001	0.369
**HR**	1.397	55.366	<0.001	0.493
**RR peak frequency**	1.451	19.185	<0.001	0.252
**HR Max-HR min**	1.558	73.009	<0.001	0.562
**SDHR**	1.354	90.846	<0.001	0.614
**VLF**	1.705	27.298	<0.001	0.324
**LF**	1.784	32.689	<0.001	0.364
**HF**	1.345	12.125	<0.001	0.175
**LF/HF**	1.774	5.753	0.006	0.092
**Skin Conductance (SC)**	1.112	119.135	<0.001	0.676

**Table 3:** Pairwise Comparisons: Mean differences (St. Dev.).

Measure	Relax vs. Stroop	Relax vs. Arithmetic	Arithmetic vs. Stroop
**Respiration Amplitude**	0.822 * (0.095)	1.581 * (0.155)	−0.760 * (0.104)
**Respiration Period**	827.565 * (86.499)	1380.723 * (114.278)	−553.157 * (88.322)
**Respiration Rate (BPM)**	−2.283 * (0.279)	−3.414 * (0.307)	1.132 * (0.196)
**BVP Amplitude**	−5.990 * (0.559)	−6.145 * (0.597)	0.155 (0.139)
**RR mean**	−68.378 * (11.398)	−66.835 * (10.747)	−1.543 (5.364)
**HR**	10.519 * (1.365)	10.985 * (1.356)	−0.466 (0.691)
**RR peak frequency**	−0.059 * (0.011)	−0.035 * (0.011)	−0.024 * (0.006)
**HR Max–HR min**	20.530 * (2.559)	29.421 * (2.991)	−8.891 * (1.794)
**SDHR**	7.247 * (0.880)	10.542 * (0.969)	−3.295 * (0.456)
**VLF**	364.900 * (45.725)	397.386 * (66.608)	−32.487 (64.606)
**LF**	628.482 * (125.608)	1047.121 * (150.396)	−418.639 * (112.210)
**HF**	644.916 * (251.945)	1045.813 * (245.665)	−400.896 * (118.022)
**LF/HF**	0.185 (0.191)	−0.379 (0.175)	0.564 * (0.138)
**Skin Conductance (SC)**	8.041 * (0.816)	9.291 * (0.751)	−1.250 * (0.223)

Based on estimated marginal means; * The mean difference is significant at the 0.05 level (Bonferroni adjusted for multiple comparisons).

**Table 4:** Stratified 10-fold Cross validation. Four learning algorithms were compared, i.e., (1) Logistic regression, (2) random forest, (3) support vector machine, and (4) Naïve Bayes. In the analysis, the classification learning algorithm was used for the classifications referring to the test used for ranking (Figure 1) [30,47,48,49].

Method	AUC	CA	F1	Precision	Recall
**Logistic Regression**	0.808	0.744	0.746	0.749	0.744
**Random Forest**	0.771	0.694	0.692	0.694	0.694
**Support Vector Machine (SVM)**	0.800	0.733	0.731	0.732	0.733
**Naive Bayes**	0.796	0.728	0.723	0.733	0.728

**AUC** (Area under the ROC curve) is the area under the classic receiver-operating curve. **CA** (Classification accuracy) represents the proportion of the examples that were classified correctly. **F1** represents the weighted harmonic average of the precision and recall (defined below). **Precision** represents a proportion of true positives among all the instances classified as positive. In our case, the proportion of a condition was identified correctly. **Recall** represents the proportion of true positives among the positive instances in our data.

**Table 5:** Parameter Estimates.

Parameter	B	Std. Error	Hypothesis Test		
			Wald Chi-Square	df	Sig.
**(Intercept)**	−59.397	6.6904	78.818	1	<0.001
**SDRR**	0.204	0.0210	94.114	1	<0.001
**Respiration Period**	0.003	0.0009	10.752	1	0.001
**HR**	0.479	0.0764	39.247	1	<0.001
**RR peak frequency**	57.614	12.3360	21.812	1	<0.001
**VLF**	0.011	0.0031	11.446	1	0.001
**(Scale)**	68.237				
Dependent Variable: HR Max–HR min (RSA)					
